# Persistent urine leakage around a suprapubic catheter: the experience of a person with chronic tetraplegia

**DOI:** 10.1038/s41394-018-0058-7

**Published:** 2018-04-04

**Authors:** Subramanian Vaidyanathan, Jerry Ward, Bakul M. Soni, Peter Hughes, Tun Oo

**Affiliations:** 1 Regional Spinal Injuries Centre, Southport and Formby District General Hospital, Town Lane, Southport, PR8 6PN UK; 2 Spinal Cord Injury Patient, Cheshire, England UK; 3 Department of Radiology, Southport and Formby District General Hospital, Town Lane, Southport, PR8 6PN UK

## Abstract

**Introduction:**

Persistent urine leakage after suprapubic cystostomy in tetraplegic subjects occurs due to shrinkage of the urinary bladder and bladder spasms. The patient’s social life is adversely affected as clothes become wet, smelly, and require frequent changing, thus increasing the workload of carers.

**Case presentation:**

A 48-year-old male sustained C-4 complete (AIS:A) tetraplegia while swimming in 2007. Suprapubic cystostomy was performed in 2009. From 2012, this patient had urine leakage around the suprapubic catheter, which became progressively more frequent. Propiverine, then oxybutynin tablets instead of propiverine, oxybutynin transdermal patches, and mirabegron in addition to oxybutynin were tried. An indwelling urethral catheter was used in addition to the suprapubic catheter to alleviate urine leakage when the bladder was undergoing spasms. This patient continued to have leakage around the suprapubic catheter. Leakage of urine was occasionally accompanied by autonomic dysreflexia. Leakage of urine caused huge amounts of extra work for carers, and family. Furthermore, leakage of urine had a significant impact on quality of life, and going out with friends and family. Bladder wall injection of Botox was performed in 2015 and in 2016, which reduced urine leakage.

**Discussion:**

Bladder wall injection of Botox to treat persistent urine leakage around the suprapubic catheter in spinal cord injury patients with suprapubic cystostomy has not been mentioned in NICE guidelines or publications indexed in PubMed. While recommending suprapubic cystostomy to subjects with tetraplegia, leakage of urine around the suprapubic catheter, which may occur sometime after suprapubic cystostomy, should be included in the conversation so that patients and carers become aware of this potential complication.

## Introduction

Management of neuropathic bladder in a person with spinal cord injury is challenging. Intermittent catheterization, urethral or suprapubic catheter drainage are commonly employed. Hunter and associates compared suprapubic catheterization to any other method of chronic bladder emptying such as intermittent and indwelling catheterization in adults in relation to complications, patient satisfaction, and health-related quality of life [1]. Suprapubic catheters were associated with a low incidence of urethral injury and stricture, but had similar rates of upper tract damage, vesicoureteral reflux, renal or bladder calculi, and symptomatic urinary tract infections compared to urethral catheters. A retrospective review of records of 133 spinal cord injury patients with urethral catheters and 46 patients with a suprapubic tubes at Long Beach Veterans Hospital, Long Beach, CA, USA revealed that leakage around the suprapubic catheter was seen in 12 of 46 (26%) patients with a suprapubic catheter [2]. Urine leakage around a suprapubic catheter is likely to occur when the catheter is blocked completely or even partially. When a patient develops a urinary tract infection, the patient may experience increased bladder spasms and consequently, leakage around the suprapubic catheter. But urine leakage due to blockage of catheter or urine leakage occurring during a urinary tract infection is transient and subsides when the underlying pathology is treated adequately. Factors to consider when a spinal cord injury patient with a suprapubic catheter develops persistent leakage of urine around suprapubic catheter are listed in Table 1. Prolonged urine leakage around a suprapubic catheter can result in complications such as soft tissue infection in the pubic region, bone infection in the pelvis, heterotopic ossification between the pubic bones, maceration and ulceration of skin in groin, perineum and ischial region [3, 4]. We describe a cervical spinal cord injury patient’s experience of persistent urine leakage which occurred about three years after undergoing suprapubic cystostomy. Urine leakage continued despite oxybutynin and mirabegron therapy. Botulinum toxin was injected submucosally into the bladder and the patient noticed some improvement. Bladder wall injection of Botox to treat urine leakage around a suprapubic catheter in patients with suprapubic cystostomy has not been described either in The National Institute for Health and Care Excellence (NICE) pathways or in scholarly publications.

**Table 1:** Factors to consider when a spinal cord injury patient with a suprapubic catheter develops persistent leakage of urine around suprapubic catheter

1. Is a suprapubic catheter of adequate size for this patient or whether increasing the size of the suprapubic catheter should be considered?
2. Does leakage around suprapubic catheter occur when the patient takes a diuretic or when the patient drinks large amounts of fluids within a short period of time?
3. How is the catheter secured?
4. Is the drainage bag kept below the suprapubic stoma, and is the tubing kept above the bag?
5. Is the catheter clamped or is a Flip Flo valve or similar device being used? A suprapubic tube in those with a neurogenic bladder is preferably not clamped, as this promotes reflux of urine colonized with bacteria.
6. Is the patient experiencing increased spasms?
7. Is the patient taking single or dual antimuscarinic drugs or a beta-3 adrenoceptor agonist alone or as add-on therapy?
8. Has the bladder capacity reduced? Is the bladder shrinking in capacity due to the use of an indwelling catheter?
9. Does the patient have regular bowel movements? Constipation can aggravate bladder spasms and urine leakage.

## Case presentation

**Fig. 1:**
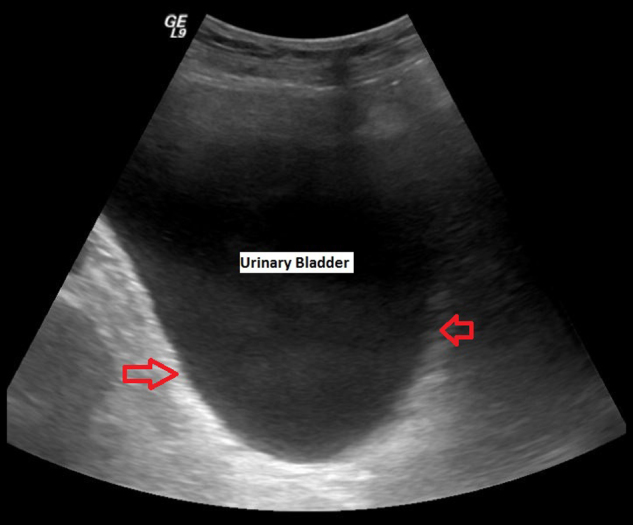
Ultrasound scan of urinary bladder performed in March 2009 (two years after spinal cord injury) showed normal bladder; no bladder wall thickening, diverticula or calculi (right and left arrows)

A 48-year-old airline pilot was hit by a rogue wave while swimming; he hit the water head/face first, causing hyperextension of his neck. He was subsequently drowned and unconscious in the water. There was no movement in both upper and lower limbs. After he was pulled from the water and resuscitated, he was breathing spontaneously initially but developed respiratory distress two days later and a tracheostomy was performed. Subsequently, examination revealed no motor or sensory function below Cervical-4. (C-4 American Spinal Injury Association Impairment Scale grade: A tetraplegia). This patient underwent anterior discectomy and fusion of C-4/C-5 vertebra five days after his injury. Post-operatively, he required a prolonged period of ventilation. Eventually, he was weaned off, the delay being due to recurrent chest infection. In 2008, this patient underwent implantation of a Synchromed II Medtronic programmable pump for intrathecal administration of baclofen to control spasticity. In March 2009, ultrasound scan showed normal kidneys with good cortical thickness; no calculi, focal scarring or hydronephrosis; normal bladder; no bladder wall thickening, diverticula or calculi (Fig. 1). This patient had an indwelling urethral catheter. The urethral catheter was getting blocked frequently and he would develop autonomic dysreflexia. Therefore, suprapubic cystostomy was performed in September 2009. During subsequent catheter changes, size of the suprapubic catheter was increased to Foley catheter size 24 CH. In the early months after suprapubic cystostomy, bladder washouts with Opti-Flo—R 50 ml were used on alternate days to maintain patency of the suprapubic catheter. Despite using bladder washouts, this patient experienced frequent blockages of the suprapubic catheter resulting in autonomic dysreflexia episodes, often requiring an admission through Accident & Emergency Department for catheter changes. In 2010 after a catheter change due to partial blockage, debris was removed from the suprapubic catheter and sent for analysis. Constituents: Calcium phosphate: 87% (Carbapatite); Magnesium ammonium phosphate: 13%. Methenamine hippurate 1 g tablet once-daily, was prescribed, along with ascorbic acid 500 mg three times daily, in an effort to make urine more acidic.

This patient developed occasional urine leakage around the suprapubic catheter from early 2012. Patency of catheter was checked; the urine bag was kept below suprapubic cystostomy stoma; the drainage tube connecting a suprapubic catheter to a urine bag was kept above the bag thus ensuring gravity drainage of urine from the urinary bladder. Urine leak around the suprapubic catheter persisted. This patient was prescribed propiverine hydrochloride 15 mg three times a day; as urine leak persisted, instead of propiverine, oxybutynin tablets (5 mg three times a day), then oxybutynin transdermal application was prescribed. Leakage of urine continued. There was occasional bleeding around the suprapubic catheter. A beta-3 adrenoceptor agonist, mirabegron, 50 mg once daily, was prescribed in addition to oxybutynin. This patient continued to have leakage around the suprapubic catheter on several occasions. The leakage of urine was occasionally accompanied with autonomic dysreflexia and a rise in blood pressure, necessitating his carers to give him nifedipine sublingually. The leakage of urine was sufficient to either completely wet all the bed clothes and pillows under his legs or soak through several incontinence sheets every evening, an awful lot of work for the carers besides extra laundry to wash. As a result of urine leakage from the suprapubic site, it became necessary to have incontinence sheets both above and below to try and prevent soiling of the bedclothes, pillows, blankets and duvets, etc. Leakage of urine around the suprapubic catheter started to become progressively more frequent, and despite very careful positioning of the catheter drainage bag whilst either seated or lying, once urine leakage started it could continue for several hours despite a suprapubic catheter being patent. Although the patient had very limited levels of sensation, the leakage of urine was occasionally accompanied by abdominal pain. The leakage of urine started to have a significant impact on quality of life, and patient’s confidence being out with his friends and family.

**Fig. 2:**
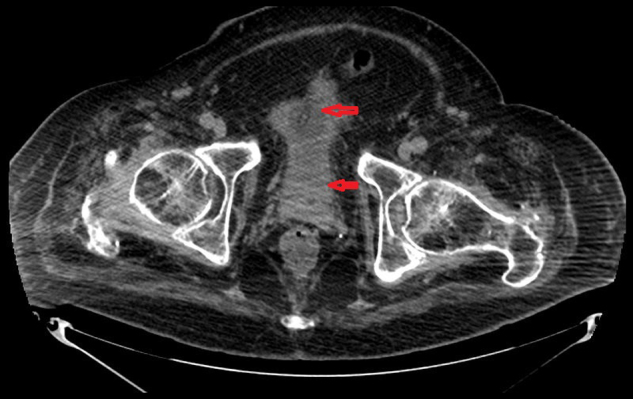
Computed tomography of pelvis performed in October 2014 (about 7 years after spinal cord injury) showed urinary bladder (bottom arrow) completely collapsed around the balloon (top arrow) of the suprapubic catheter

**Fig. 3:**
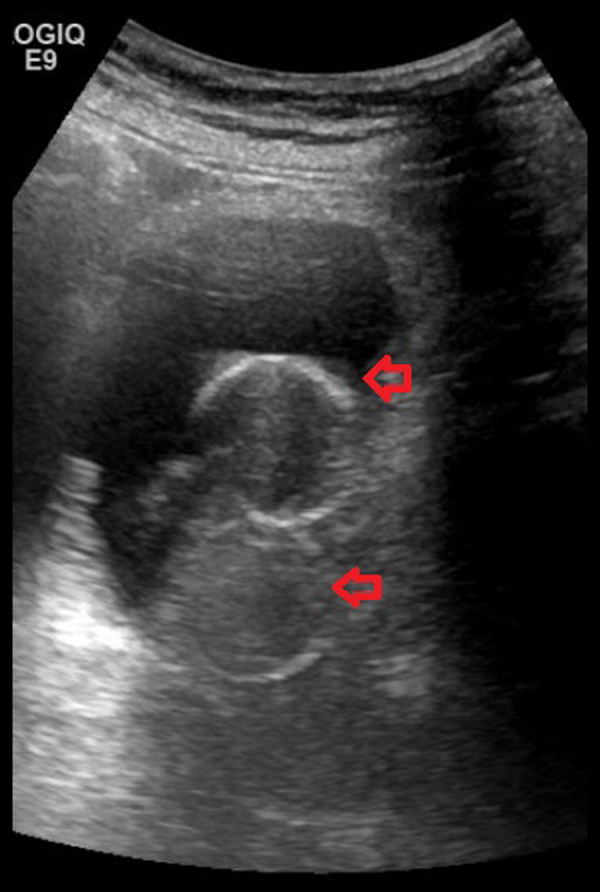
Ultrasound scan of urinary bladder performed in August 2015 (eight years after spinal injury) showed presence of both urethral catheter balloon (bottom arrow) and suprapubic catheter balloon (top arrow) within the small capacity urinary bladder

In October 2014, Computed Tomography of pelvis showed the urinary bladder to be completely collapsed around the balloon of a suprapubic catheter (Fig. 2). An indwelling urethral catheter was used in addition to a suprapubic catheter, to try and alleviate leakage when the bladder was undergoing spasms. In March 2015, ultrasound scan of the urinary tract revealed normal kidneys. Attempts to fill the bladder failed due to leakage of urine around the suprapubic catheter. Urine leakage around the suprapubic site continued. In August 2015, ultrasound scan of the urinary tract confirmed presence of both urethral and suprapubic catheter balloons within the urinary bladder (Fig. 3). This scan ruled out misplacement of urinary catheters for persistent leakage of urine around a suprapubic catheter.

Because of persistent leakage of urine from suprapubic site, this patient started to reduce considerably his fluid intake. The problems became so severe that the carers were being required to work much more through early evening and frequently through the night, which was not sustainable. The constant leakage, checking of the catheter site region, repositioning of the catheter drainage bag, etc. and replacement of dressing around the suprapubic site, as well as changing all the incontinence sheets started to become too much for the carers. During the evening time in particular, quality of life was reduced significantly. Even with severely reduced intake of fluids, once the bladder spasms started and urine leakage occurred, leakage of urine around a suprapubic catheter would continue for several hours. Video-urodynamics was not performed as there was leakage of fluid around suprapubic catheter when the bladder was filled with saline. In September, 2015, following regular urology outpatient clinic visit cystoscopy revealed small contracted bladder. This patient was admitted, and arrangements were made for cystoscopy. Botox 300 units diluted to 10 ml was injected, 0.5 ml at 20 sites in the bladder. Following Botox injection in September 2015, leakage of urine became rather less frequent, but still continued on occasion. The patient always used a “Cath-Grip” to secure the catheter to his abdominal area, and since October 2016 he had stopped swapping which side of his abdomen the Cath-Grip was placed on when doing routine changes. For some reason, when a suprapubic catheter was routed across the right-hand side of patient’s abdomen, the leakage of urine was much more frequent. A small clamp is used occasionally to artificially block the catheter and allow the bladder muscle to expand; normally for about one hour at a time. Despite all these efforts, it was felt necessary to repeat the Botox injection after a period of just more than one year. In October 2016, Botox, 300 units, diluted to 20 ml, and one ml was injected submucosally in 20 sites in the urinary bladder. After the repeat operation in October 2016, leakage of urine steadily improved. Careful positioning of the drainage bag was still very important. If there were any restrictions in the tube, such as small kinks, or, if the tube was routed “up and over” patient’s leg or knee, the leakage of urine would still occur. However, because of the increased confidence, the patient started feeling much more able to get on with his life, and go out and about. It is likely that a further injection of botulinum toxin will be required in early 2018. This will be predicted by leakage/bypassing once more becoming more frequent.

## Discussion

The gold-standard treatment for neurogenic detrusor over-activity is anticholinergic medication and intermittent self-catheterization [5]. Long-term indwelling catheterization in the bladder and associated chronic inflammation can make the bladder low compliant in spinal cord injured patients. Probable mechanisms of vesical low compliance are given in Table 2. Bladder spasms and low compliant bladder predispose to urine leakage around suprapubic catheter in spinal cord injury patients. Persistent urine leakage can affect the personal life of an individual with spinal cord injury. This patient has been leading an active social life. Therefore, this patient preferred to persevere with suprapubic catheter drainage despite urine leakage and try adjuvant drug therapy to mitigate urine leakage.

**Table 2:** Possible causative factors for low compliance bladder in persons with spinal cord injury

1. Long standing infection and stimulation due to foreign body such as indwelling Foley catheter, may result in structural changes in the bladder wall such as fibrosis
2. Myogenic origin: Spinal cord injury patients have denervated muscles, which lead to increased collagen and hypertrophy of smooth muscles fibers
3. Neurogenic origin: Hypertrophy of the detrusor muscle of neuropathic bladder could induce a hyperactivity of nerve and muscle cell. Low compliance can be induced by autonomous contraction of newly produced cholinergic nerve fibers following injury of parasympathetic nerves [8]

### Management of persistent urine leakage

Persistent urine leakage occurring in a patient with low compliant bladder and bladder spasms is managed by prescribing anti-muscarinic drugs either alone or in combination. In patients, who do not respond to anti-muscarinic drugs or when the response is sub-optimal, beta-3 adrenoceptor agonist, Mirabegron may be useful to relax the bladder and reduce urine leakage. Mirabegron has been tried as an add-on therapy also. NICE pathways state that bladder wall injection with botulinum toxin type A may be offered to adults with spinal cord injury and with symptoms of an overactive bladder and in whom antimuscarinic drugs have proved to be ineffective or poorly tolerated [6]. Injection of Botulinum toxin type A into the urinary bladder of spinal cord injury patients, who have had a suprapubic cystostomy and who leak urine around a suprapubic catheter is not mentioned in this pathway.

Bladder wall injection of Botulinum toxin has been linked to life-threatening adverse effects on tissues at a distance from the injection site, following its diffusion throughout the body. Muscle weakness, asthenia and constipation have been reported [7]. Cho and Kim [5] also stated that the use of onabotulinum toxin A in patients with high cervical lesions above T1 carries the risk of developing muscular weakness in the respiratory muscles. Therefore, Botulinum toxin injection should be used only when the bladder spasms are not adequately controlled by antimuscarinic drugs prescribed singly or in combination, and beta-3 adrenoceptor agonist, used alone or in addition to anti-muscarinic drug. This patient received oxybutynin and mirabegron and even then, the leakage of urine around a suprapubic catheter persisted; therefore botulinum toxin injection into bladder wall was used, which resulted in a significant decrease in urine leakage. Video-urodynamics was not performed as urine would leak around a suprapubic catheter when the urinary bladder was filled.

SF-Qualiveen score (0–5) was calculated at various time points in this patient. Lower the score, lesser the disability is; higher the score, greater is the disability due to urinary bladder problems. Soon after suprapubic cystostomy, the score was 3.0. Before first Botox injection, (August 2015), SF-Qualiveen score increased to 3.875. Following first Botox injection, (October 2015), the score decreased slightly to 3.75. In November 2016, soon after second Botox Injection, the SF-Qualiveen score was high at 3.875. About one year after second Botox injection (September 2017), the score decreased to 3.125.

SF-Qualiveen score indicated that soon after suprapubic cystostomy, when leakage of urine did not occur, the score was still high at 3.0. In other words, suprapubic cystostomy did not lead to total abatement of the bladder problems in this patient. When leakage of urine occurred, the bladder problems affected adversely how this person with tetraplegia lived and understandably, the SF-Qualiveen score increased. This patient required two sittings of Botox injections and a time lapse of a few months after second Botox injection for the adverse impact of urine leakage upon day-to-day life to diminish. Even after two courses of Botox injections, the effect of urine leakage continued to make a significant impact on the life of this tetraplegic person, as reflected by a SF-Qualiveen score of 3.125 (range: 0–5).

## Learning points

Persistent and significant leakage of urine around suprapubic catheter affecting quality of life is a potential complication of suprapubic cystostomy in spinal cord injury patients. While recommending suprapubic cystostomy to tetraplegic subjects, leakage of urine around a suprapubic catheter, which may occur sometime after suprapubic cystostomy, should be included in the conversation so that the patient and the carers become aware of this potential complication.
